# Preoperative diagnosis of pancreatic body and tail cyst: a case of gastric bronchogenic cyst and literature review

**DOI:** 10.1093/jscr/rjae658

**Published:** 2025-05-03

**Authors:** Han Duan, Congcan Zhao, Yunbo Tan, Liquan Jin

**Affiliations:** Dali University of Clinical Medicine School, Dali, Yunnan 671000, China; Dali University of Clinical Medicine School, Dali, Yunnan 671000, China; 1st Department of General Surgery, The First Affiliated Hospital of Dali University, Dali, Yunnan 671000, China; 1st Department of General Surgery, The First Affiliated Hospital of Dali University, Dali, Yunnan 671000, China

**Keywords:** bronchogenic cyst, gastric, diagnosis, treatment, literature review

## Abstract

Bronchogenic cyst is a condition arising from congenital abnormalities in the respiratory system. It can be classified into intrapulmonary, mediastinal, and ectopic types depending on its location. Ectopic types are rare, and diagnosis typically depends on pathological examination. This paper presents a case of a female patient admitted to our hospital with abdominal pain. Preoperative imaging revealed a mass in the tail of the pancreas, which was surgically excised. Postoperative pathology confirmed the diagnosis of a gastric bronchogenic cyst. Based on the patient’s diagnosis and treatment, it is recommended that the diagnosis of gastric bronchogenic cysts be supported by auxiliary examinations, such as imaging, with final confirmation achieved through postoperative histopathology, as suggested by a review of the relevant literature. Surgical treatment is the preferred approach for gastric bronchogenic cysts.

## Introduction

Bronchogenic cyst is a rare congenital malformation of the tracheobronchial bud, originating from the primitive foregut during early embryonic development [1]. Ectopic bronchogenic cysts are most commonly found in the lungs and mediastinum, with rare occurrences in the abdomen and even rarer cases in the stomach. These cysts typically do not exhibit specific signs on computed tomography (CT) or magnetic resonance imaging (MRI). As a result, the misdiagnosis rate for this condition can range from 40% to 60%, with the final diagnosis relying on pathological examination [2]. It is challenging to accurately diagnose gastric bronchogenic cysts preoperatively, as most are identified after surgery [3]. Imaging studies play a key role in the diagnosis and differential diagnosis of bronchogenic cysts. This article presents a case of a 53-year-old patient with a gastric bronchogenic cyst identified in the pancreatic tail on preoperative imaging and reviews the relevant literature.

## Case presentation

**Figure 1:**
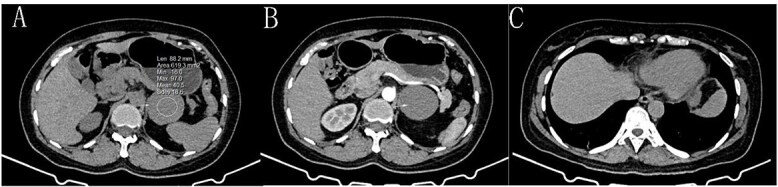
A: Gastric bronchogenic cyst on a plain CT scan (→), with a CT value of approximately 40.5 HU. B: The CT-enhanced arterial phase shows no enhancement of the cyst (→). C: A postoperative plain CT scan shows that the cyst has been successfully removed.

A 53-year-old female presented with intermittent upper abdominal distension and pain, accompanied by radiating pain in the lower back. She denied experiencing diarrhea, nausea, vomiting, black stools, or bloody stools. There were no symptoms of chills, fever, chest tightness, shortness of breath, palpitations, or dizziness. One day later, she sought treatment at the Emergency Department of the First Affiliated Hospital of Dali University. The patient had a history of angina pectoris for over six months and had been on long-term oral medications, including atorvastatin, metoprolol, and aspirin. She had no history of other gastrointestinal diseases and denied any other chronic illnesses, infectious diseases, trauma, surgery, or family history. She is married with children of an appropriate age. A thorough physical examination was performed. Her vital signs were stable, with no apparent abnormalities in the cardiopulmonary assessment. The abdomen was flat and soft, with no visible gastrointestinal or peristaltic waves. Palpation revealed tenderness in the upper abdomen, without rebound pain or palpable abdominal mass. Abdominal percussion and auscultation showed no obvious abnormalities. An emergency CT scan of the upper and middle abdomen revealed: [1] an undetermined soft tissue mass in front of the left kidney, [2] a small fat density focus (10 × 6 mm) beneath the capsule of the right lower lobe of the liver, and [3] possible cholecystitis with stones. An emergency hepatobiliary, pancreatic, and spleen ultrasound revealed a cystic mass in the retroperitoneal pancreatic tail (suggestive of a pancreatic cyst). Symptomatic treatment, including pain relief, was provided in the outpatient department, but the patient’s symptoms showed no significant improvement. She was subsequently admitted to the hospital due to a suspected pancreatic space-occupying lesion, possibly a pancreatic cyst. After admission, she underwent further auxiliary examinations. Results indicated a hypersensitivity C-reactive protein level greater than 10 mg/L, a white blood cell count of 12.76 × 10^9^/L, and a neutrophil percentage of 82.2%. No significant abnormalities were found in routine urine analysis, liver and kidney function tests, electrolytes, infectious disease screening, or coagulation function. An enhanced CT scan of the upper and middle abdomen revealed a round soft tissue density shadow in the left retroperitoneum, measuring approximately 5.1 × 5.5 cm, with clear boundaries. The enhanced scan showed no enhancement. The tail of the pancreas was slightly compressed, and the left adrenal gland was compressed and displaced (Fig. 1). The radiologist suggested the presence of a left retroperitoneal mass, of undetermined nature, and gallbladder plication. Based on the patient’s medical history and auxiliary examinations, and after a discussion of the condition, a comprehensive diagnosis was made, including: (1) left retroperitoneal mass (possibly a pancreatic cyst), (2) gallbladder stones with chronic cholecystitis, and (3) coronary atherosclerotic heart disease. Following consultation with the family, it was decided that the patient would undergo mass resection surgery on the third day after admission.

**Figure 2:**
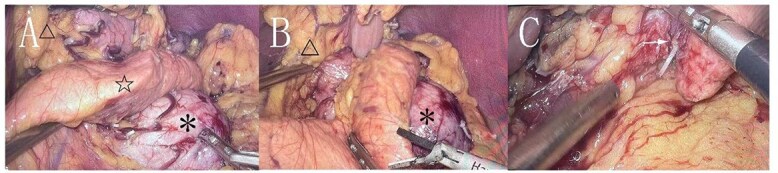
Cyst (*), pancreas (☆), lesser curvature of the stomach (△). A: The cyst originates from the lesser curvature of the stomach, located posterior to the pancreas. B: The cyst is adjacent to the lesser curvature of the stomach. C: The cyst after separation from the surrounding tissue (→ indicating the broken end).

**Figure 3:**
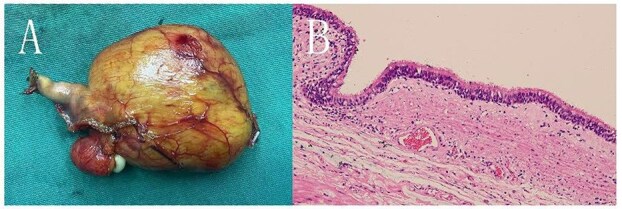
A: The cyst seen with the naked eye, displaying a clear boundary. B: HE staining under a light microscope (10 × 20 magnification) showing the cyst wall tissue, with ciliated columnar epithelium, foam cells, and lymphocytes visible.

During surgery, a lump was identified behind the tail of the pancreatic body, with no apparent adhesion or invasion of the surrounding area. The pancreatic tail was completely freed and suspended along with the splenic artery and vein, revealing that the mass originated from the lesser curvature of the stomach (Fig. 2). After fully dissociating the mass, the gastric tissue was separated from it along the lesser curvature, and the mass was completely removed from the surface of the left renal vein. The mass, visible to the naked eye, measured approximately 5 × 6 cm (Fig. 3), and yellow viscous content was observed upon incision. Postoperative pathological examination revealed a retroperitoneal mass. The fibrous cyst wall tissue was lined with ciliated columnar epithelium, with numerous foam cells and lymphocyte infiltration, suggesting a diagnosis of bronchogenic cyst (Fig. 3). The postoperative diagnosis included gastric bronchogenic cyst, gallbladder stones with chronic cholecystitis, and coronary atherosclerotic heart disease. The patient received postoperative symptomatic treatment, including antiinfection therapy and fluid replacement. A follow-up abdominal CT scan six days after surgery showed no obvious residual cysts (Fig. 1C), and the patient was discharged.

## Discussion

The exact pathogenesis of bronchogenic cysts remains unclear. These cysts develop as masses due to abnormal respiratory system development during the embryonic period. Based on their location, they can be classified as mediastinal, intrapulmonary, or ectopic types. Compared to the rare extrathoracic and subdiaphragmatic bronchogenic cysts, gastric bronchogenic cysts are even more uncommon [4]. Bronchogenic cysts are infrequent, with an incidence ranging from 1 in 68 000 to 1 in 42 000, primarily affecting the lungs or mediastinum [2]. However, in rare cases, they can involve the stomach wall [5]. In terms of symptoms, some patients may present with an asymptomatic mass, while others may experience chest pain, cough, dyspnea, or fever [6]. In this case, the patient was diagnosed with a cyst during imaging due to abdominal pain.

It is challenging to make a definitive diagnosis of gastric bronchogenic cyst before surgery, though imaging can assist in the process. On CT, bronchogenic cysts typically appear as unilocular, ovate lesions with well-circumscribed smooth, or lobulated borders. They are usually described as homogenous hypoattenuating lesions without enhancement, and calcification may sometimes be present in the cyst wall [7]. In this case, the cyst had a CT value of 40.5 HU, slightly lower than that of the liver, and ultrasound indicated cystic imaging. Gastric bronchogenic cysts must be differentiated from other conditions such as cystic lymphoma, cystic mesothelioma, cystic teratoma, gastrointestinal cysts, gastrointestinal stromal tumors, adrenal tumors, and pancreatic tumors. Histopathological examination is necessary to confirm the diagnosis of bronchogenic cyst. These cysts are characterized by a lining of respiratory tissue, ciliated cuboidal, or columnar epithelium, which may be pseudostratified, and contain thick, mucoid material secreted from this lining. Goblet cells, lymphoid aggregates, glands, and/or cartilage may also be associated with the respiratory epithelium [8]. In this case, the patient had a unique anatomical feature, with the cyst located at the tail of the pancreatic body. It is critical during surgery to completely free the pancreatic body and tail, while carefully protecting the splenic and left renal veins from potential damage. The bronchogenic cyst developed near the lesser curvature of the stomach, close to the pancreatic tail. If the tail of the pancreas is not fully released during surgery, there is a risk of misdiagnosis as a mass of the pancreatic tail, which could lead to unnecessary pancreatectomy and splenectomy. This could result in the unnecessary removal of vital organs, placing a significant physical and financial burden on the patient. To avoid such outcomes, subjective judgments should be avoided during surgery. Instead, the surrounding tissues should be completely freed, and a thorough search should be conducted to identify the cyst’s source. This approach can help preserve vital organs and ensure complete removal of the lesion.

**Figure 4:**
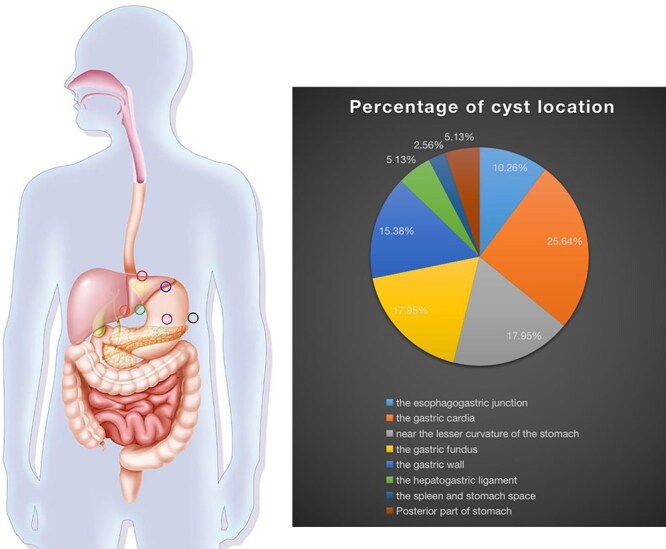
Location and percentage distribution of cysts.

Due to the limited number of cases reported globally, optimal therapeutic strategies for the clinical management of gastric bronchogenic cysts have not yet been clearly established. According to the cases reported in the literature (Table 1), gastric bronchogenic cysts are categorized into eight general locations (Fig. 4). Of these, 10.26% are found at the gastroesophageal junction, 25.64% at the cardia, and 17.95% near the lesser curvature of the stomach. The fundus of the stomach accounts for 17.95%, the gastric wall for 15.38%, the hepatogastric ligament for 5.13%, the spleen and stomach space for 2.56%, and the posterior part of the stomach for 5.13%. Most patients in the reported cases underwent surgical resection of gastric bronchogenic cysts. The literature suggests that early surgical resection, even in asymptomatic cases, is recommended to confirm the diagnosis, relieve symptoms, and prevent complications. Surgical resection remains the most effective method to achieve a definitive diagnosis [9]. Given the shorter surgical time and reduced intraoperative bleeding, along with the potential for minimizing economic burden and postoperative pain, laparoscopic resection is advised for the diagnosis and treatment of gastric bronchogenic cysts. Robotic surgery has also been utilized, though its widespread adoption is limited due to its high cost. Postoperative monitoring is essential for optimal patient care. Patients should undergo regular follow-up after surgery. The prognosis following surgical resection of bronchogenic cysts is generally favorable, as no cases of recurrence or metastasis have been reported in the literature we reviewed.

**Table 1:** The documents searched for ‘bronchogenic cyst AND gastric’; in the PubMed database from 2000 to March 2024 are as follows.

Author	Report type	Title	Location of cyst
Ma YQ, Bi XR, Zhan WP, Ma YT.	Letter	Cyst of bronchogenic origin between the abdominal aorta and the lesser curvature of the stomach	Between the abdominal aorta and the lesser curvature of the stomach
Lin Z, Cao Q.	Case report	Gastric bronchogenic cyst	The posterior wall of the fundus near the cardia
Shang F, Xu Y, Jiao J, Lian C.	Review	Prone to misdiagnose the gastric bronchogenic cyst: a case report and literature review	Cardia and corpus
Qian W, Xu G.	Review	Gastric fundus bronchogenic cyst with elevated CA72–4: a case report and literature review	The submucosal eminence near the gastric fundus
Liu T, Li J.	Case report	Gastric cardia bronchogenic cyst with prolonged belching	In the gastric cardia
Qian JW, Qian ZH, Wu YY, Liu PF.	Case report	Bronchogenic cyst of stomach: a case report	Close to the gastric cardia wall, but not connected to the gastric cavity
Terayama M, Kumagai K, Kawachi H, *et al.*	Case report	Optimal resection of gastric bronchogenic cysts based on anatomical continuity with adherent gastric muscular layer: a case report	Adjacent to the cardia of the stomach
Ma P, Gao F, Zhu W, Cao Y.	Case report	Bronchial-origin gastric cyst: a rare case report	In the gastric wall in the upper part of the gastric body
Lou F, Chen Q, Hu H.	Case report	Gastric bronchogenic cyst in a young woman	Close to the gastric fundus wall, but not connected to the gastric cavity
Wang HB, Wang SL, Yu JW.	Case report	A gastric bronchogenic cyst preoperatively misdiagnosed as a gastrointestinal stromal tumor	The lesser gastric curvature near the gastroesophageal junction
Murakami T, Shimizu H, Yamazaki K, *et al.*	Case report	Intra-abdominal ectopic bronchogenic cyst with a mucinous neoplasm harboring a *GNAS* mutation: a case report	On the lesser curvature of the stomach
Kihara T, Sugihara T, Ikeda S, *et al.*	Case report	Gastric bronchogenic cyst: a rare congenital cystic lesion of the stomach	Between the left lobe of the liver and the lesser curvature of the gastric wall
Erbenová A, Placrová B, Špůrková Z, Horák P.	Review	Bronchogenic cyst of gastric cardia—case report and literature review	In the gastric cardia wall
Sun B, Wang AK, Chen H, *et al.*	Review	Bronchogenic cyst of the stomach: a case report and literature review	A spleen and stomach space
He WT, Deng JY, Liang H, Xiao JY, Cao FL.	Case report	Bronchogenic cyst of the stomach: a case report	In the posterior wall of the fundus
Xiao J, Zhang R, Chen W, Wu B.	Case report	Ectopic bronchogenic cyst of the gastric cardia considered to be a gastrointestinal stromal tumor before surgery: a case report	On the side of the lesser curvature, near the cardia
Han WG, Xue HD, Pan WD.	Case report	Bronchogenic cyst of stomach: report of one case	The lesser curvature side of the gastric body, near the cardia
Ruiz Molina I, Landauro Comesaña C, Solís García E, *et al.*	Case report	Gastric bronchogenic cysts: an unusual location	In the hepatogastric ligament
Chhaidar A, Ammar H, Abdessayed N, *et al.*	Case report	Large bronchogenic cyst of stomach: a case report	In the gastric cardia
Tonouchi A, Kinoshita T, Sunagawa H, *et al.*	Case report	Bronchogenic cyst at esophagogastric junction treated by laparoscopic full-thickness resection and hand-sewn closure: a case report	On the anterior wall of the esophagogastric junction
Tu C, Zhu J, Shao C, Mao W, *et al.*	Review	Gastric bronchogenic cysts: a case report and literature review	In the cardia of the stomach 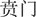
Gou Y, Wang Y, Fang H, *et al.*	Case report	Bronchogenic cyst in the hepatogastric ligament masquerading as an esophageal mesenchymal tumor: a case report	In the hepatogastric ligament
Sun L, Lu L, Fu W, Li W, Liu T.	Case report	Gastric bronchogenic cyst presenting as a gastrointestinal stromal tumor	In the fundus of the stomach close to the esophagogastric junction
Kurokawa T, Yamamoto M, Ueda T, *et al.*	Case report	Gastric bronchogenic cyst histologically diagnosed after laparoscopic excision: report of a case	Close proximity to the gastric cardia, and it protruded into the lesser omentum cavity
Yang X, Guo K.	Case report	Bronchogenic cyst of stomach: two cases report and review of the English literature	The fundus and cardia of the stomach under the diaphragm
Seddik H, Adioui T, Rouibaa F, *et al.*	Case report	Gastric bronchogenic cyst presenting as a submucosal mass: a case report	In the juxtacardial stomach
Ubukata H, Satani T, Motohashi G, *et al.*	Case report	Intra-abdominal bronchogenic cyst with gastric attachment: report of a case	Attached to the lesser curvature of the stomach
Jiang L, Jiang L, Cheng N, Yan L.	Case report	Bronchogenic cyst of the gastric fundus in a young woman	The gastric fundus
Tan KK, Nandini CL, Ho CK.	Case report	A case of gastric bronchogenic cyst in Singapore with multiple intrigues	The posterior wall of the stomach that was resting on the anterior surface of the pancreas
Shibahara H, Arai T, Yokoi S, Hayakawa S.	Case report	Bronchogenic cyst of the stomach involved with gastric adenocarcinoma	At the lesser curvature of the stomach
Sato M, Irisawa A, Bhutani MS, *et al.*	Case report	Gastric bronchogenic cyst diagnosed by endosonographically guided fine needle aspiration biopsy	Compressing the gastric lumen approximately 2 cm from the cardia
Gillion JF, Lagneau M, Balaton A, *et al.*	Case report	Bronchogenic cyst of the juxtacardiol stomach mimicking a stromal tumor associated with a bronchogenic cyst of the subdiaphragmatic esophagus	The posterior part of the stomach
Lee SH, Park DH, Park JH, *et al.*	Case report	Endoscopic mucosal resection of a gastric bronchogenic cyst that was mimicking a solid tumor	The gastric wall
Liang MK, Marks JL.	Comment	Congenital bronchogenic cyst in the gastric mucosa	Within the gastric mucosa far from the fundus of the stomach
Wang WY, Jiang LL, Liu WP, Zhang WY.	Case report	Bronchogenic cyst in gastric wall	Posterior wall of gastric body
Melo N, Pitman MB, Rattner DW.	Case report	Bronchogenic cyst of the gastric fundus presenting as a gastrointestinal stromal tumor	The gastric fundus
Rubio CA, Orrego A, Willén R.	Case report	Bronchogenic gastric cyst: a case report	In the gastric mucosa
Song SY, Noh JH, Lee SJ, Son HJ.	Case report	Bronchogenic cyst of the stomach masquerading as benign stromal tumor	In the lesser curvature side just below the gastroesophageal junction

## Conflict of interest statement

None declared.

## Funding

None declared.
